# Nasal cavity geometry of healthy adults assessed using acoustic rhinometry

**DOI:** 10.1016/S1808-8694(15)31386-0

**Published:** 2015-10-17

**Authors:** Adriana de Oliveira Camargo Gomes, Ana Claudia Martins Sampaio-Teixeira, Sergio Henrique Kiemle Trindade, Inge Elly Kiemle Trindade

**Affiliations:** 1 MSc at the Graduate Program on Rehabilitation Sciences at Hospital de Reabilitação de Anomalias Craniofaciais (HRAC-USP). PhD student at the Graduate Program on Rehabilitation Sciences at HRAC-USP, Physiology Laboratory, Bauru-SP; 2 PhD at the Graduate Program on Rehabilitation Sciences at HRAC-USP. Physiologist at the Physiology Laboratory, HRAC-USP, Bauru-SP; 3 Specialist on Otorhinolaryngology, PhD student at the Graduate Program on Otorhinolaryngology at Faculdade de Medicina (FMUSP). MD, otorhinolaryngologist at Hospital do Servidor Público Estadual-SP, Hospital Estadual de Bauru-SP and Hospital das Clínicas-FMUSP-SP; 4 Full Professor - Department of Biologic Sciences at Faculdade de Bauru-USP and at the Physiology Laboratory at HRAC-USP, Bauru-SP. Hospital de Reabilitação de Anomalias Craniofaciais Universidade de São Paulo

**Keywords:** nasal cavity, acoustic rhinometry, reference values

## Abstract

Acoustic rhinometry (AR) has been used as a specific test for nasal patency.

**Aim:**

this study aimed to set the reference values for nasal cavity cross-section geometry in healthy adults through AR.

**Study design:**

this is a clinical prospective study.

**Materials and method:**

thirty volunteers (14 males and 16 females) without signs of nasal obstruction and aged between 18 and 30 years were enrolled in this study. They were assessed before and after being treated topically with a nasal vasoconstrictor drug. Their nasal cross-sectional areas were measured at the three dips of the rhinogram, corresponding respectively to the nasal valve (CSA1), the anterior (CSA2), and the posterior (CSA3) region of the inferior and middle turbinate.

**Results:**

the mean areas (±SD) for 60 nasal cavities before nasal vasoconstriction were: 0.54±0.13cm2 (CSA1), 0.98±0.31 cm2 (CSA2), and 1.42±0.44cm2 (CSA3). After vasoconstriction, the mean values of the three segments analyzed were significantly larger (p<0.05). Gender was not a statistically significant variable.

**Conclusion:**

The nasal cross-sectional areas obtained for adults may be used for control purposes when studying patients with nasal obstruction, in conjunction with the nasal volume values previously reported by our group.

## INTRODUCTION

Instrumental methods were developed in the past few decades to objectively check nasal patency and allow for further clinical finding confirmation. The most frequently used technique is rhinomanometry, in which patency is estimated based on nasal airflow resistance, from the simultaneous verification of flow and transnasal pressures generated during breathing at rest. Despite its validity, nasal airflow resistance has its limitations, the most important being it is flow-dependent^1^, ^2^. In order to overcome such limitation, Warren^3^ introduced a modification to conventional rhinomanometry to estimate the minimum nasal cross-sectional area, which in terms of airflow resistance measurement offers the advantage of not being flow-dependent. It was shown that in adults over 18 years of age values under 0.40 cm2 are indicative of nasal obstruction^1^, ^3^.

Nasal cross-sectional area also began to be measured by acoustic rhinometry from the study conducted by Hilberg et al.^4^. The test is based on the measurement of the reflected sound waves (echo) that emerge from the nasal cavity in response to introduced sound waves. It allows sequential measurements of nasal cavity segments, from the nostrils to the choanae, thus facilitating the on-site identification of constrictions that contribute to nasal airflow resistance. Volumes for various regions in the nose can be assessed, enabling the analysis of the topographic profiles of each nasal airway^4^, ^5^, ^6^, ^7^, ^8^. This test represents a step forward in relation to rhinomanometry as proposed by Warren^3^, once it allows the assessment of only the narrowest cross-section segment, usually the nasal valve, although it required more cooperation from the patient.

**Chart 1:**
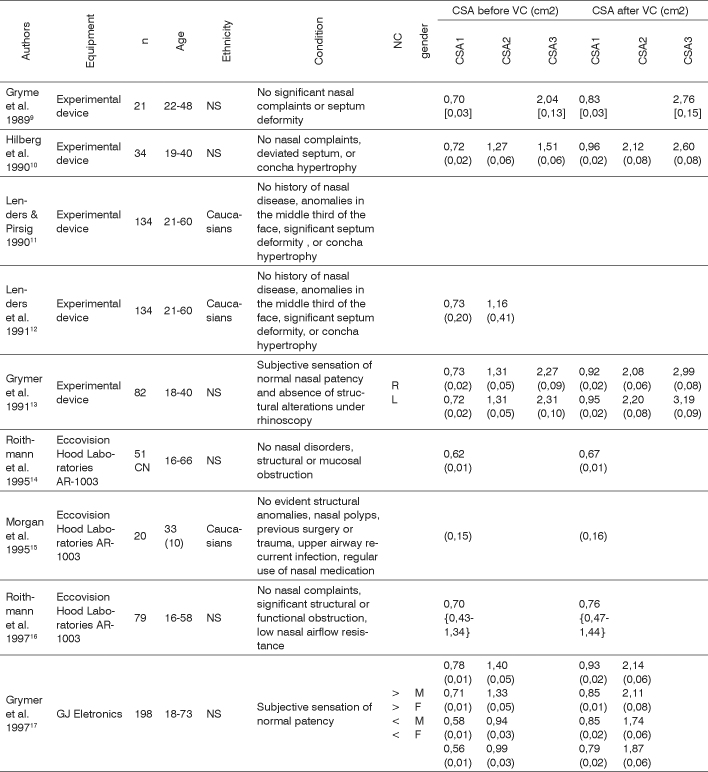
Nasal cross-sectional areas (CSA) reported in the literature for adults without evidences of nasal obstruction, before and after vasoconstriction (VC).

Today acoustic rhinometry is broadly recognized as a specific test for nasal patency. Normal cross-sectional area values have been reported by many authors^9^, ^10^, ^11^, ^12^, ^13^, ^14^, ^15^, ^16^, ^17^, ^18^, ^19^, ^20^, ^21^, ^22^, ^23^, ^24^, ^25^, whose findings are summarized on Chart 1. Nonetheless, due to factors such as ethnic, weather, and laboratory-related differences, local reference values must be determined, as stressed by Hilberg and Pedersen^7^ and Roithman^26^ more recently.

Therefore, in the study conducted in our laboratory, we first defined the volumes of three areas in the nasal cavity (valve, concha, and nasopharynx) of adults without evidences of nasal obstruction^27^. This paper aims to present values for nasal cross-sectional areas of three segments - nasal valve, anterior and posterior portions of the inferior and middle turbinates - gathered from a sample of healthy individuals, so that these values can be used as reference in studies conducted upon populations with specific diseases and to better understand the outcomes of therapeutic and surgical interventions. Additionally to that, differences related to gender and variations caused by the use of nasal vasoconstrictor drugs are analyzed.

## MATERIALS AND METHOD

### Materials

Sixty nasal cavities were analyzed from a sample of 30 adult volunteers without evidences of nasal obstruction (14 males and 16 females aged between 18 and 30 years), after they signed a free informed consent form. Participants were selected based on answers given in a questionnaire designed to identify past and present signs and symptoms of nasal obstruction and on nasal airflow verification using Glatzel’s mirror, as described in the first paper of the series^27^. Twenty-four individuals were excluded from the original sample of 54, as they had history of structural or functional nasal anomalies, nasal trauma, recurring respiratory infection, regular use of vasoconstrictor drugs, mouth breathing, or clearly reduced nasal airflow in the mirror test. No formal calculations were done on the number of participants.

This study was approved by the Research Ethics Committee of our institution under permit 070/2002-UEP-CEP.

### Equipment and testing principles

**Figure 1:**
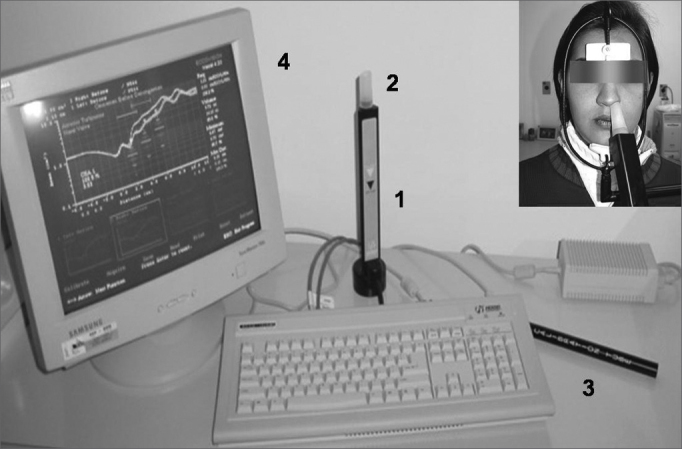
Acoustic rhinometer (Eccovision, Hood Laboratories): instrumentation to check nasal cavity cross-sectional areas; 1=sound tube; 2= nasal adapter; 3=calibration tube 4=computer monitor showing a rhinogram. See patient in position for data acquisition.

Tests were conducted using an Eccovision Acoustic Rhinometer (HOOD Laboratories), which consists of a sound source (loudspeaker) distally positioned in relation to a 24 cm tube equipped with a microphone for acquisition in its proximal portion. The rhinometry tube is placed against one of the nostrils; a sound wave generated by the loudspeaker propagates through the tube, passes through the microphone and enters the nasal cavity. Variations in the cross-sectional area, i.e., constrictions reducing cavity lumen, reflect the sound waves back into the rhinometry tube. Pressure signals sensitize the microphone and are amplified and digitized. A computer equipped with specific software is used to analyze the signals. The system is shown on Fig. 1.

**Figure 2:**
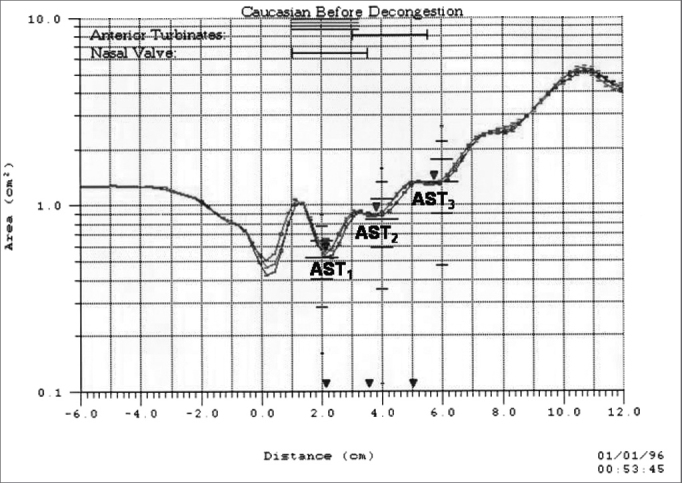
Rhinogram: area-distance graph produced by acoustic rhinometry, depicting the nasal cavity cross-sectional areas measured in the sites corresponding to the nasal valve (CSA1), the anterior (CSA2) and the posterior (CSA3) portions of the middle and lower nasal turbinates.

Nasal cross-sectional areas are calculated from the echo intensity values. Distance to site of constriction is calculated based on wave speed and time of echo feedback. The data is converted into an area-distance function and presented on the computer monitor in the shape of a graph - the rhinogram - in which areas are shown in a semi-logarithmic scale on axis Y (in cm^2^) and distances on the axis X (in cm) as seen in Fig. 2. The rhinometer generates 10 sound pulses in rapid succession (approximately one every 0.5 seconds) and at each test the software calculates the average of the cross-sectional areas acquired for the 10 cycles. The system allows measurements in the entire nasal cavity, and of the right and left sides independently.

### Procedure and analyzed variables

Three measurements were carried out for each nasal cavity, before and 10 minutes after administering 5 drops of a nasal vasoconstrictor drug (0.1% xylometazoline) on each nostril after nasal hygiene. The values used in our analysis are the averages for the three measurements conducted in three technically acceptable curves for each condition.

As discussed in detail in the first paper of the series^27^, in order to minimize error in the measurements as the ones resulting from ambient temperature variation and external noise, all tests were carried out in the same room, at a relatively stable ambient temperature and with noise levels never above 60dB, and after waiting for 30 minutes for the patients to adapt to the local conditions. The rhinometry tube was always placed in a parallel position in relation to the nasal dorsum. The contact area between the nasal adapter tip and the nasal cavity end was sealed with neutral gel so as to avoid sound losses. The chins and foreheads of the participants were supported to keep their heads stable during examination as they were placed parallel to the floor, as seen in Fig. 1. The tests were conducted during voluntary nasal breathing suspension after exhaling. The patients were told to remain with their mouths closed, without swallowing or moving their tongues during data acquisition, so as to prevent breathing and swallowing from interfering with the measurements and harming the quality of the rhinograms. We were also careful enough not to deform the participants’ nostrils and consequently their nasal valves. The rhinometer used in the study offers an adapter that is supposed to be lightly touched against the nostril, instead of introduced into it as is the case for the olive-shaped adapters^9^, ^10^, ^11^, ^12^, ^13^, which by itself precludes nasal valve deformation. Patients had their spectacles removed so as to remove external pressure from the nose. The equipment was calibrated at the beginning of each day.

For analysis purposes, we considered the nasal cross-sectional areas (in cm^2^) obtained in the second dip of the area-distance curve which corresponds to the nasal valve region (CSA1), in the third dip which corresponds to the anterior portion of the middle and inferior turbinate (CSA2), and in the fourth dip which corresponds to the posterior portion of the middle and inferior turbinate (CSA3), as seen in Fig. 2. The first dip, seen from distance zero and equivalent to the nostril, was not considered for analysis^28^.

### Data analysis

Considering that variable CSA follows a normal distribution18, the group results are expressed as averages ± standard deviation. Student’s T test was used to analyze the statistical significance of the differences between independent samples (males vs. females). Statistical significance difference for related paired samples (before vs. after sing nasal vasoconstrictor) was assessed through Student’s T test. Significance was assigned when p<0,05.

## RESULTS

Table 1 shows the average values for CSA1, CSA2 and CSA3 for the 30 right nasal cavities and 30 left nasal cavities analyzed in this study, acquired before and after nasal vasoconstrictor drug administration. Sample size is not uniform for the three samples as, in some cases, it was not possible to measure CSA2 or CSA3 as their respective dips in the rhinogram were not identified.

**Table 1:** Nasal cross-sectional areas (CSA1, CSA2 and CSA3) measured through acoustic rhinometry in 60 nasal cavities of 30 adults without evidences of nasal obstruction, according to gender and cavity side (right - R; left - L), before and after nasal vasoconstriction (VC)

CSA (cm^2^)		Before VC			After VC	
	R	L	R and L	R	L	R and L
MALES CSA^1^ (valve)	0,59±0,19	0,56±0,13#	0,57±0,16	0,62±0,15	0,57±0,13#	0,60±0,14
	(n=14)	(n=14)	(n=28)	(n=14)	(n=14)	(n=28)
CSA^2^ (anterior portion of the turbinates)	1,06±0,37	0,95±0,35#	1,00±0,36	1,63±0,34	1,47±0,22#	1,55±0,29
	(n=11)	(n=14)	(n=25)	(n=9)	(n=9)	(n=18)
CSA^3^ (posterior portion of the turbinates)	1,41±0,51	1,46±0,56#	1,43±0,53	2,05±0,32	2,16±0,36#	2,10±0,33
	(n=13)	(n=14)	(n=27)	(n=13)	(n=11)	(n=24)
FEMALES CSA^1^ (VALVE)	0,51±0,10	0,52±0,1#	0,51±0,10*	0,55±0,09	0,53±0,13#	0,54±0,10*
	(n=16)	(n=16)	(n=32)	(n=16)	(n=16)	(n=32)
CSA^2^ (anterior portion of the turbinates)	0,96±0,28	0,97±0,25#	0,96±0,27*	1,29±0,36	1,51±0,33#	1,37±0,36*
	(n=16)	(n=15)	(n=31)	(n=15)	(n=8)	(n=23)
CSA^3^ (posterior portion of the turbinates)	1,36±0,29	1,44±0,43#	1,40±0,36*	2,07±0,77	1,89±0,39#	1,99±0,57*
	(n=15)	(n=15)	(n=30)	(n=7)	(n=10)	(n=17)

Average ± standard deviation

n = number of cavities analyzed

# not a statistically significant difference (right side vs. left side)

* not a statistically significant difference (males vs. females, analyzed to the right and left)

Statistical analysis showed no significant differences between the CSA averages verified in the right (R) and left (L) sides before and after vasoconstrictor administration. Therefore, both sides were considered as independent cavities and new measurements were taken and averages calculated for all 60 nasal cavities (right and left), as also seen in Table 1. Gender statistical differences were analyzed. CSA averages were not statistically significant differences between males and females before and after vasoconstriction.

Table 2 shows the average CSA values for the whole group, i.e., with males and females considered together and their respective observed percent variation after vasoconstrictor administration. Statistical analysis showed that vasoconstriction resulted in comparatively higher measured values, and larger percent variation for variables CSA2 and CSA3.

**Table 2:** Nasal cross-sectional areas (CSA1, CSA2 and CSA3) measured through acoustic rhinometry in 60 nasal cavities of 30 adults of both genders without evidences of nasal obstruction, before and after vasoconstriction (VC).

CSA (cm^2^)	Before VC	After VC	Percent Variation
CSA ^1^	0,54±0,13	0,56±0,13^S^	4%
(valve)	(n=60)	(n=60)	
CSA^2^	0,98±0,31	1,45±0,34^S^	
(anterior portion of the turbinates)	(n=56)	(n=41)	48%
CSA ^3^	1,42±0,44	2,06±0,45^S^	45%
(posterior portion of the turbinates)	(n=57)	(n=41)	

Average ± standard deviation

n = number of cavities analyzed

S p<0.05: statistically significant difference (before vs. after VC)

## DISCUSSON

The recommendations for technical specifications and standard operating procedures from the European Rhinological Society^7^ include, among others, the definition of standard values for nasal areas and volumes. Following this guideline, this study aimed at defining reference values for cross-sectional areas (CSA) of specific segments of the nasal cavity of normal adult individuals, thus completing the data provided in a previously published paper on the reference values for nasal volumes^27^.

Two methodological issues deserve special attention. Firstly, the group of 30 individuals analyzed in this study were conveniently selected from a population of 54 apparently health individuals based on subjective reports of normal nasal patency sensation and no history of nasal functional and anatomical disorders, as also seen in control groups enrolled by other authors^17^, ^20^, ^21^, ^23^. When comparing the values acquired from our sample to the 0.35cm^2^ indicated as normal by Hilberg and Pedersen7, we verified that only one of the 60 cavities analyzed had an area smaller than the value defining adequate nasal patency, strongly indicating that our sample was indeed made up by subjects without nasal obstruction. It is important to mention that in this case specifically the subject’s contralateral cavity had an area of 0.78cm^2^, thus compensating for the likely structural or functional unilateral obstruction and sensation of adequate nasal patency. Secondly, the tests were carefully performed to take into account and control the variables pointed by other authors^7^, ^14^, ^29^ that could impact measurement accuracy and reproducibility, such as ambient temperature, external noise, rhinometry tube position, sound losses, head position, nostril deformation, equipment calibration, and interferences from breathing and swallowing. By doing so in other studies carried out at our laboratory, we observed variation in cross-sectional area rhinometry measurements ranging between 6% and 9%^30^.

Let us now analyze the nasal cross-sectional areas acquired in this study and their differences in comparison to those reported in the literature for normal adults, as seen in Table 1. Firstly, let us compare our results to those gathered by Corey et al.^18^, used as reference by the manufacturer of the Eccovision Acoustic Rhinometer. In our study, the averages of the areas measured for all 60 cavities were 0.54±0.13cm2 (CSA1), 0.98±0.31cm2 (CSA2), and 1.42±0.44cm2 (CSA3), with no differences between genders. These values, obtained for the group as a whole, were quite close to those reported by Corey et al.18, respectively 0.52±0.12cm2 (CSA1), 0.83±0.24cm2 (CSA2), and 1.31±0.42cm2 (CSA3). The same trend was observed for the values obtained after vasoconstriction.

For simplification purposes, we will now compare our CSA1 values (before vasoconstriction) - the standard measurement in all studies published on this subject - to the ones reported in other papers. The difference between average CSA1 values was never beyond 10%, when comparing our data set to that of various authors^18^, ^19^, ^23^, ^24^, ^25^, which ranged between 0.52cm^2^ and 0.59cm^2^, thus validating the results published in this paper. The papers cited above are quite recent, and two used the same equipment employed in our study. In all others^14^, ^15^, ^16^, ^17^, ^20^, ^21^, ^22^, the average CSA1 values ranged between 0.60cm^2^ and 0.78cm^2^. This difference may be attributed to the fact that most studies - specially the ones done longer ago - used olive-type nasal adapters which ae known to introduce nasal cavity deformation, thus leading to overestimated cross-sectional area measurements^14^, ^29^. Other factors that may explain the observed differences are the characteristics and the size of the analyzed sample, the type of equipment employed, and even the differences related to factors that may potentially introduce error in the measurements as mentioned before^7^, ^27^. These findings stress the relevance of defining reference values for each laboratory.

In some of the rhinograms it was not possible to identify the dips related to CSA2 and CSA3, more specifically after vasoconstriction. One may speculate that this resulted from absence of constrictions along the nasal cavity after the valve that could modify sound reflection and thus create a dip in the rhinogram, similarly to what would occur in a straight tube. We cannot, however, discard the possibility that this was a technical artifact.

In terms of gender differences, differently from what was observed in the larger volumes seen in males in the areas close to the valve and turbinates^27^, we found that CSA1, CSA2 and CSA3 were not statistically different for males and females. This allowed the analysis of all 60 nasal cavities in one large group, as seen in Table 2, and the consideration of reference values for both genders, an approach not recommended for nasal volumes.

Finally, we should comment on the results obtained under vasoconstriction. This procedure is performed to identify structural changes in the nasal fossae, considering that the functional effect of the mucosa is removed. Similarly to what was seen for nasal volumes^27^, we observed a significant increase in the cross-sectional area of the three segments analyzed when under nasal vasoconstriction. The effect was more evident on CSA2 and CSA3 than on CSA1, thus confirming that the nasal valve is proportionally less susceptible to mucosal status variation than the turbinates.

## CONCLUSION

This study used acoustic rhinometry to determine the reference values for nasal cross-sectional areas to be used, for comparison purposes, in the analysis of adults with functional and/or anatomical nasal obstruction. The findings we gathered reinforce the relevance of rhinometry as a valuable tool to enhance the assessment of nasal patency and better understand nasal and respiratory physiology.
